# First molecular characterization of *Dirofilaria Immitis* in Cuba

**DOI:** 10.1186/s12917-023-03803-0

**Published:** 2023-11-17

**Authors:** Lisset Roblejo-Arias, Cristian Díaz-Corona, Elianne Piloto-Sardiñas, Adrian A. Díaz-Sánchez, Zbigniew Zając, Joanna Kulisz, Aneta Woźniak, Sara Moutailler, Dasiel Obregon, Angélique Foucault-Simonin, Belkis Corona-González, Alejandro Cabezas-Cruz

**Affiliations:** 1 Direction of Animal Health, National Center for Animal and Plant Health, Carretera de Tapaste y Autopista Nacional, Apartado Postal 10, San José de las Lajas, Mayabeque, 32700 Cuba; 2 Laboratoire de Santé Animale, ANSES, INRAE, Ecole Nationale Vétérinaire d’Alfort, UMR BIPAR, Maisons-Alfort, France; 3 University of Saskatchewan, Saskatchewan, Canada; 4 Department of Biology and Parasitology, Medical University of Lublin, Radziwiłłowska 11st, Lublin, 20-080 Poland; 5 School of Environmental Sciences, University of Guelph, Guelph, ON Canada

**Keywords:** Zoonosis, *Dirofilaria Immitis*, Canine filariae, Dog, Cuba

## Abstract

**Background:**

Dirofilarioses are widespread diseases caused by mosquito-borne nematodes of the family Onchocercidae, genus *Dirofilaria*. The major etiologic agent of canine dirofilariosis in the American continent is the zoonotic parasite *Dirofilaria immitis*. Existing reports of filarioid nematodes in Cuba are based solely on morphological and immunological analysis which do not allow unambiguous identification and/or direct detection of causal agents.

**Results:**

Here we present the molecular characterization of filarioid nematodes found in a dog in Cuba. Based on the molecular and phylogenetic analysis of the 5.8S-ITS2-28S region and *cox1* gene fragments, the worms were unambiguously classified as *D. immitis*. Sequence analysis showed high identity of the gene fragments in this study with others previously obtained from *D. immitis* found in dogs, wolfs and jackals but also from mosquito vectors of *D. immitis*.

**Conclusions:**

Further studies are guarantee to better understand the epidemiological impact of canine dirofilariosis in Cuba as well as the competence of different species of culicid mosquitoes as vectors of *Dirofilaria* in the country.

**Supplementary Information:**

The online version contains supplementary material available at 10.1186/s12917-023-03803-0.

## Background

Dirofilariosis is a mosquito-borne disease with worldwide distribution caused by nematodes of the genus *Dirofilaria*, family Onchocercidae. Filariae of the *Dirofilaria* genus can infect wild and domestic animals of several orders including Rodentia, Artiodactyla, Perissodactyla, Carnivora, Lagomorpha, Edentata, and Primates [1]. About 60–70 mosquito species of the Culicidae family are considered as intermediate hosts and/or vectors of *Dirofilaria* worldwide [2].

*Dirofilaria repens* and *Dirofilaria immitis* are two *Dirofilaria* species of special interest due to their negative impact on company animals (i.e., dogs and cats) as well as their zoonotic potential [3, 4]. The final hosts of both parasite species are mainly canine predators including dogs, wolfs (*Canis lupus*), foxes (*Vulpes vulpes*), and jackals (*Canis aureus*), but cats and weasels (*Mustela nivalis*), can also be infected [5]. Originally, dirofilariasis was considered a disease of strict veterinary importance [6]. However, it is currently recognized as an emerging zoonosis [6]. Clinical symptoms of dogs infected by *D. immitis* include respiratory distress, epistaxis, haemoptysis, ascites, exercise intolerance, and anorexia [2, 7]. However, most animals infected with *D. immitis* are asymptomatic or display no abnormalities in different laboratory tests [8]. Several parasitological, serological, and molecular tests are available for the detection of *D. immitis* with varying levels of sensitivity and specificity [9–11]. Microscopic analysis tests, such as blood smears from peripheral veins and capillaries [12], and the modified Knott test (concentration method) are used to detect circulating microfilariae [13] and considered quick and low-cost. However, the specificity and sensitivity of filarioid species identification with these tests are low sensitivity for people with no or poor experience in the diagnosis [14].

The diagnostic tests based on molecular methods have high sensitivity, and allow for filarioid differentiation [14, 15]. In addition, molecular methods can detect low parasitemia in infected animals given a more realistic picture of the parasite prevalence [14, 15]. The Polymerase Chain Reaction (PCR) is currently being recommended as a species-specific test in the detection of *D. immitis* [10, 16], however the combination of different diagnostic tests is an important element in epidemiological studies addressing the detection of *D. immitis* [17, 18].

Several species of *Dirofilaria* including *D. acutiuscula*, *D. striata*, *D. immitis* and *D. repens* have been reported as causing infectation in dogs in the Americas [19–21], but *D. immitis* is the most important causative agent of canine dirofilariosis in the continent [22]. In North America, the prevalence of *D. immitis* in domestic dogs has been estimated to range from 1 to 12% [19], while in Central and South America, the prevalence is much higher, reaching 42% in cities on the Gulf Coast of Mexico, 63.2% in the Caribbean, 45% in Brazil, and 74% in Argentina [20, 23].

*Dirofilaria immitis* was reported in Cuba in 1977 and in the 80’s several reports were published on the presence of this parasite in the country [24–27]. In all these reports, the identification of the parasite was achieved by morphological and immunological analysis [24–27]. In addition, in 1992, the presence of *D. immitis* was reported in a dog using a coagglutination assay [28]. However, to the authors’ knowledge, no molecular characterization of *D. immitis* strains circulating in Cuba is available. Therefore, the aim of our study was to describe the morphological and molecular characterization of filarioid nematode collected from an infected dog in Cuba. Sequence and phylogenetic analyses showed to the presence of a *D. immitis* strain similar to that reported in other canids and mosquito vectors of *D. immitis*.

## Results

### Clinical examination and microscopic evaluation

**Fig. 1:**
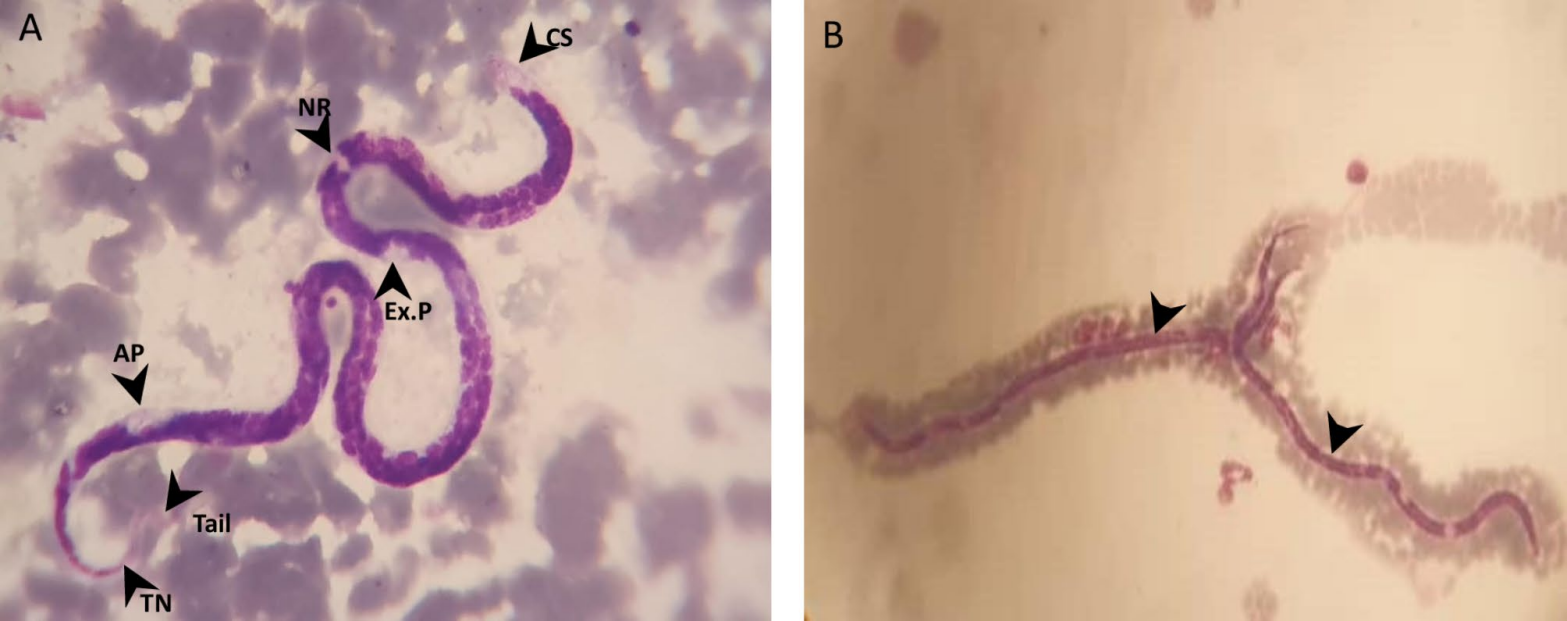
Microscopic observation of microfilaria. **(A)** Microfilaria of the genus *Dirofilaria* identified on Giemsa-stained thin blood smears from a dog. The morphological marks show the position of several structures. CS: cephalic space; NR: nerve ring; Ex.P: excretory pore; AP: anal pore; TN: terminal nucleus. (1000× magnification). **(B)** Two distended microfilariae of the genus *Dirofilaria* (arrow) (100× magnification)

No clinical sign or symptoms indicative of diseases were observed in the dog. However, microscopic observation of Giemsa-stained blood smear revealed the presence of Microfilariae. The morphological examination of the microfilariae showed unsheathed microfilaria and structures such as the cephalic space, nerve ring, excretory pore, anal pore, and the terminal nucleus at the tail were identified (Fig. 1), which allowed assigning the worm to the genus *Dirofilaria*. The dog had never been tested for heartworm or administered heartworm prophylactics.

### PCR results

**Fig. 2:**
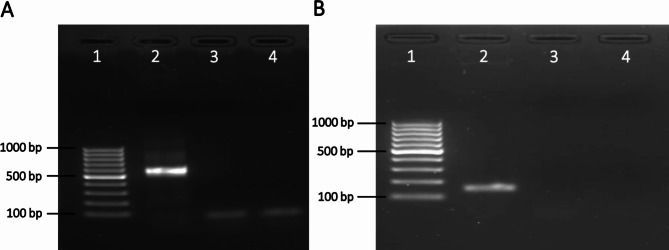
Gel electrophoresis of PCR products amplified using filarioid-specific primers. **(A)** Amplification of PCR products using filarioid-specific 5.8S-ITS2-28S region primers on a 1.5% agarose gel. Lane 1: GeneRuler 100 bp Plus DNA Ladder; lane 2: dog sample DNA; lane 3: negative control; and lane 4: water control. (B) Amplification of PCR products using primers for the cytochrome oxidase subunit 1 (*cox1*) fragment specific to *D. immitis* on a 2% agarose gel. Lane 1: GeneRuler 100 bp Plus DNA Ladder; lane 2: dog sample DNA; lane 3: negative control; and lane 4: water control. The original photograph of the gel electrophoresis is available as Supplementary Figure S1

The pan-filarioid primer pair targeting the 5.8 S ribosomal RNA gene and internal transcribed spacer 2 (5.8S**-**ITS2-28S) rRNA region amplified a PCR amplicon at size corresponding to *D. immitis* DNA (542 bp) (Fig. 2A). On the other hand, a 150 bp PCR product was amplified using the *D. immitis cox1*-specific primers (Fig. 2B). The nucleotide sequences originating from the 5.8S**-**ITS2-28S region obtained in the present study was submitted to GenBank (accession number OQ784647). Due to short fragment size, GenBank did not allow the deposition of the amplified *cox1* fragment, which can be provided upon request.

### Phylogenetic analysis

**Fig. 3:**
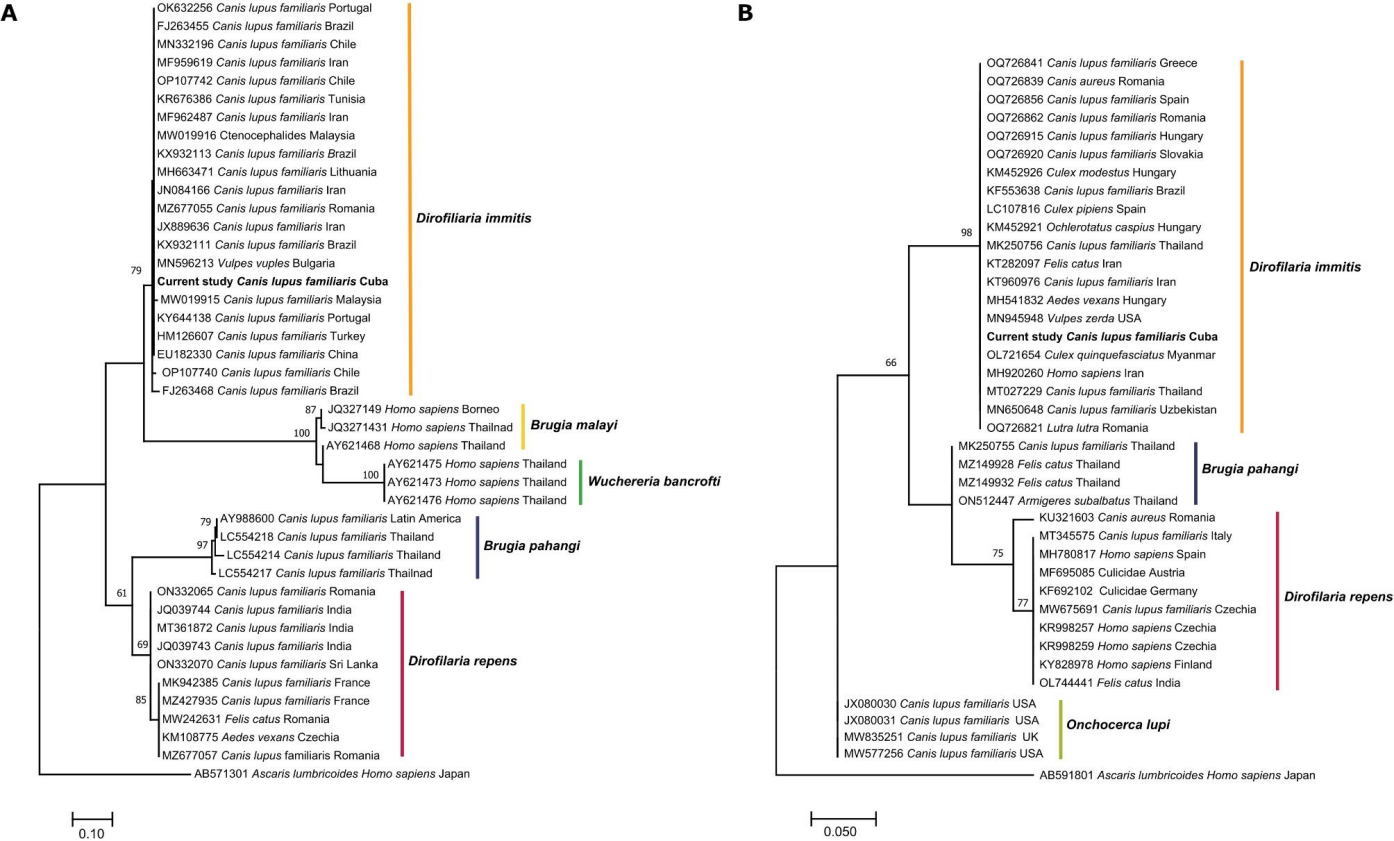
Phylogenetic tree of selected representatives of Onchocercidae. (**A**) Phylogram representing analysis of the 5.8S-ITS2-28S rRNA region. The evolutionary history was inferred with maximum likelihood method and Tamura 3-parameter (T92) model. Analysis contains sequences uploaded from GenBank (with accessions numbers and host) and obtained in the current study (in bold). Bootstrap values are represented as per cent of internal branches (1000 replicates), values lower than 60 are hidden. The tree is drawn to scale, with branch lengths measured in the number of substitutions per site. *Ascaris lumbricoides* (AB571301) was used to root the tree. **(B)** Phylogram representing analysis of *cox1* gene. The evolutionary history was inferred with maximum likelihood method and Tamura 3-parameter (T92) model. Analysis contains sequences uploaded from GenBank (with accessions numbers and hosts) and obtained in the current study (in bold). Bootstrap values are represented as per cent of internal branches (1000 replicates), values lower than 60 are hidden. The tree is drawn to scale, with branch lengths measured in the number of substitutions per site. *Ascaris lumbricoides* (AB591801) was used to root the tree

Phylogenetic analysis of 5.8S**-**ITS2-28S rRNA region (Fig. 3A) and *cox1* gene fragment (Fig. 3B) placed sequences obtained in the current study together with other *D. immitis* sequences available in GenBank. Obtained fragment of 5.8S**-**ITS2-28S rRNA region clustered together with previously reported sequences of *D. immitis* collected from its primary hosts, i.e., canine *Canis lupus familiaris* (Malaysia MW019915, Brazil KX93211, Iran JX889636), and *Vulpes vulpes* (Bulgaria MN596213). Analysed sequence showed also similarity to microfilariae of this species collected from insects of Ctenocephalides family (MW019916). Similarly, sequence of *cox1* gene clustered with other *D. immitis* collected from canine, e.g., *C. lupus familiaris* (Thailand MT027229, Slovakia OQ726920), *Vulpes zerda* (USA MN945948); its primary vector *Culex quinquefasciatus* (Myanmar OL721654) and accidental host, e.g., human (Iran MH920260).

## Discussion

Dirofilariosis is an emerging parasitic infection of growing concern in the world [29]. Canine heartworm, caused by *D. immitis,* has a wide distribution in Latin America [30], and the Caribbean region in Turk and Caicos Islands [31], Curacao [32], Grenada [33], St Kitt [34], Haiti [35], Dominican Republic [36] and Puerto Rico [37] and in island environments outside America [34]. In Cuba, the first reports were made from 1977 to 1992 using microscopic examination and coagglutination assay [24–28], and as far as the authors know, no other reports in animals or humans have been published.

The gold standard test for the diagnosis of microfilariae is the microscopic examination of blood smear stained with Giemsa or hematoxylin and eosin [38]. However, microscopic examination has low sensitivity, cannot clearly discriminate among closely related species of filarioid nematodes (e.g., *D. immitis*, *D. repens*, and *D. reconditum* or *Brugia malayi* and *Brugia pahangi*), and requires considerable expertise [39]. Here, we provided morphological and molecular evidence of the presence of *D. immitis* in a dog from Cuba. Until now, no studies combining morphological and molecular diagnosis of canine microfilariae in Cuba have been published.

Sequence analysis of 5.8S**-**ITS2-28S rRNA and *cox1* gene fragments showed high identity with sequences obtained previously from *D. immitis* parasites found in dogs, wolfs and jackals [5, 6] as well as mosquito vectors of this nematode [2]. Low genetic diversity between sequences of *D. immitis* obtained in the current study and other sequences uploaded to GenBank data BLAST is supported by previously published reports [40–42] and may confirm stable maintenance and circulation of this parasite between its vectors and hosts in the environment. Moreover, this phenomenon can be also explained by potential infection of *Wolbachia* sp., common bacterial endosymbiont of *D. immitis* influencing its survival and reproduction rate [43] but additionally reducing genetic polymorphism in *Wolbachia*-infected species [44].

## Conclusions

Our findings justify a better characterization of the epidemiology of *Dirofilaria* infection in dogs, wild animals, and humans in Cuba. The role of different species and/or strains of culicid mosquitoes as vectors of *D. immitis* in the country should also be evaluated. These studies are important due to the risk of introduction of non-endemic *Dirofilaria* species or strains in the country. Additionally, veterinarians should be aware of the possibility that *D. immitis* infection might be an emerging condition with zoonotic potential in Cuba.

## Methods

### Clinical examination

A one-year-old intact male mixed-breed dog born in Cuba and that had not left the country, underwent a routine examination by a local veterinary. The clinical inspection included anamnesis and clinical evaluation. The veterinarian performed a thorough physical examination that included several aspects such as body temperature recording, evaluation of mucous membranes, pulse assessment, hydration status, capillary refilling time, and manually checking the animal for skin lesions and tick infestation. Moreover, the evaluation involved observing the dog’s behavior, gait, and coordination, along with a neurologic examination the respiratory system was examined for symptoms. During the examination, the veterinarian searched for any signs or symptoms of dirofilariosis. The anamnesis was performed to gather information regarding the dog’s medical history, including previous illnesses, surgeries, and treatments. Any abnormalities or concerns detected were documented and addressed accordingly.

### Sample collection

A blood sample was aseptically drawn from the jugular vein using sterile Vacutainer needles and EDTA tubes (Becton-Dickinson Vacutainer Systems, Franklin Lakes, NJ, USA) and maintained at 4 °C within 24 h of blood collection until further analysis.

### Microscopic evaluation

A thin blood smear was prepared, stained with Giemsa solution (Merck, Boston, MA, USA) and examined under a light microscope (Carl Zeiss Microscopy GmbH, Jena, Germany) at final magnifications of 100X and 1000X. Morphological identification was performed using keys previously reported [45].

### DNA extraction and polymerase chain reaction

Total nucleic acid was extracted from 300 µL of EDTA-anticoagulated blood sample using the Wizard® Genomic DNA Purification kit (Promega, Madison, WI, USA), according to the manufacturer’s instructions. A negative control was set in which DNA extraction was carried out using 300 µL phosphate-buffered saline (PBS) (Sigma, St. Louis, MO, USA) instead of blood. The quantitative and qualitative evaluation of nucleic acid extraction was determined using a Colibri Microvolume Spectrophotometer (Titertek-Berthold, Pforzheim, Germany). The extracted nucleic acid sample was stored at ­20 °C until further use.

PCR reactions were carried out with species-specific primers to amplify fragments of 5.8S-ITS2-28S rRNA [46] and cytochrome c oxidase subunit 1 (*cox1*) [47] (Table 1). Each PCR reaction consisted of 1X Phusion HF Buffer (Thermo Scientific), 200 µM dNTPs, 0.5 µM each primer, 0.02 U/µL Phusion DNA polymerase (Thermo Scientific) and 5 µL of DNA solution in a total volume of 20 µL. The program for 5.8S-ITS2-28S rRNA fragment amplification consisted of a denaturing step at 98 °C for 30 s and 35 cycles of denaturing (10 s at 98 °C), annealing (30 s at 60 °C) and extension (30 s at 72 °C), a final extension (10 min at 72 °C) and a soak at 4 °C. The program for *cox1* gene fragment amplification consisted in a denaturing step at 95 °C for 60 s, 35 cycles of denaturing (20 s at 95 °C), annealing (20 s at 60 °C) and extension (40 s at 72 °C), a final extension (10 min at 72 °C) and a soak at 4 °C. An Eppendorf Mastercycler Nexus Gradient (Eppendorf, Hamburg, Germany) was used for the PCR reactions. The molecular size of the PCR amplicons was examined on a 1.5% agarose gel for 5.8S-ITS2-28S rRNA and 2% agarose gel for *cox1* gene, using a GeneRuler 100 bp Plus DNA Ladder (Thermo Scientific). Ethidium bromide was used as DNA-staining agent, visualized under UV light. Amplicon sequencing was commissioned to Eurofins MWG Operon (Ebersberg, Germany).

**Table 1:** Primer sequences used in this study

Primer names	Primer sequences	Targeted DNA	References
DIDR-F1 DIDR-R1	5′ AGTGCGAATTGCAGACGCATTGAG 3′ 5′ AGCGGGTAATCACGACTGAGTTGA 3′	5.8S-ITS2-28S rRNA region	[46]
DI-F1 DI-R1	5′ ATTGGGTGCCCCTGAAATGG 3′ 5′ CCCTCTACACTCAAAGGAGGA 3′	*cox1* gene	[47]

### Phylogenetic analysis

In order to determine species identity and genetic diversity of filarioid nematodes collected in the current study, obtained sequences of 5.8S**-**ITS2-28S rRNA and *cox1* gene were trimmed manually in BioEdit software v.7.2 [48], analyzed in GenBank database through the National Center for Biotechnology Information (NCBI; Bethesda, MD) and searched against Basic Local Alignment Search Tool (BLAST) (https://blast.ncbi.nlm.nih.gov/Blast.cgi, accessed on 17 April 2023). Next, sequences were aligned using MUSCLE algorithm available in MEGA X. Phylogenetic trees were constructed using maximum likelihood (ML) method and Tamura 3-parameter (T92) model, according to the lowest Bayesian Information Criterion (BIC) and Akaike information criterion corrected for small sample sizes (AICc) [49]. Reliability of internal branches was assessed using the bootstrapping method with 1000 replicates.

### Electronic supplementary material

Below is the link to the electronic supplementary material.

Supplementary Material 1: Supplementary Figure S1 original photograph of the gel electrophoresis of Filarioid PCR products. (A) Amplification of PCR products using filarioid-specific 5.8S-ITS2-28S region primers on a 1.5% agarose gel. Lane 1: GeneRuler 100 bp Plus DNA Ladder; lane 2: dog sample DNA; lane 3: negative control; and lane 4: water control. (B) Amplification of PCR products using primers for the cytochrome oxidase subunit 1 (cox1) fragment specific to D. immitis on a 2% agarose gel. Lane 1: GeneRuler 100 bp Plus DNA Ladder; lane 2: dog sample DNA; lane 3: negative control; and lane 4: water control. Lanes 5 to 10 in both panels (A and B) are not relevant to the current study.

## Data Availability

All data generated or analysed during this study are included in the article.
