# Supplementary figures and images for: First molecular characterization of Dirofilaria Immitis in Cuba
Source: BMC Vet Res. 2023 Nov 17;19:239. doi: 10.1186/s12917-023-03803-0 (PMC10655431; doi:10.1186/s12917-023-03803-0)

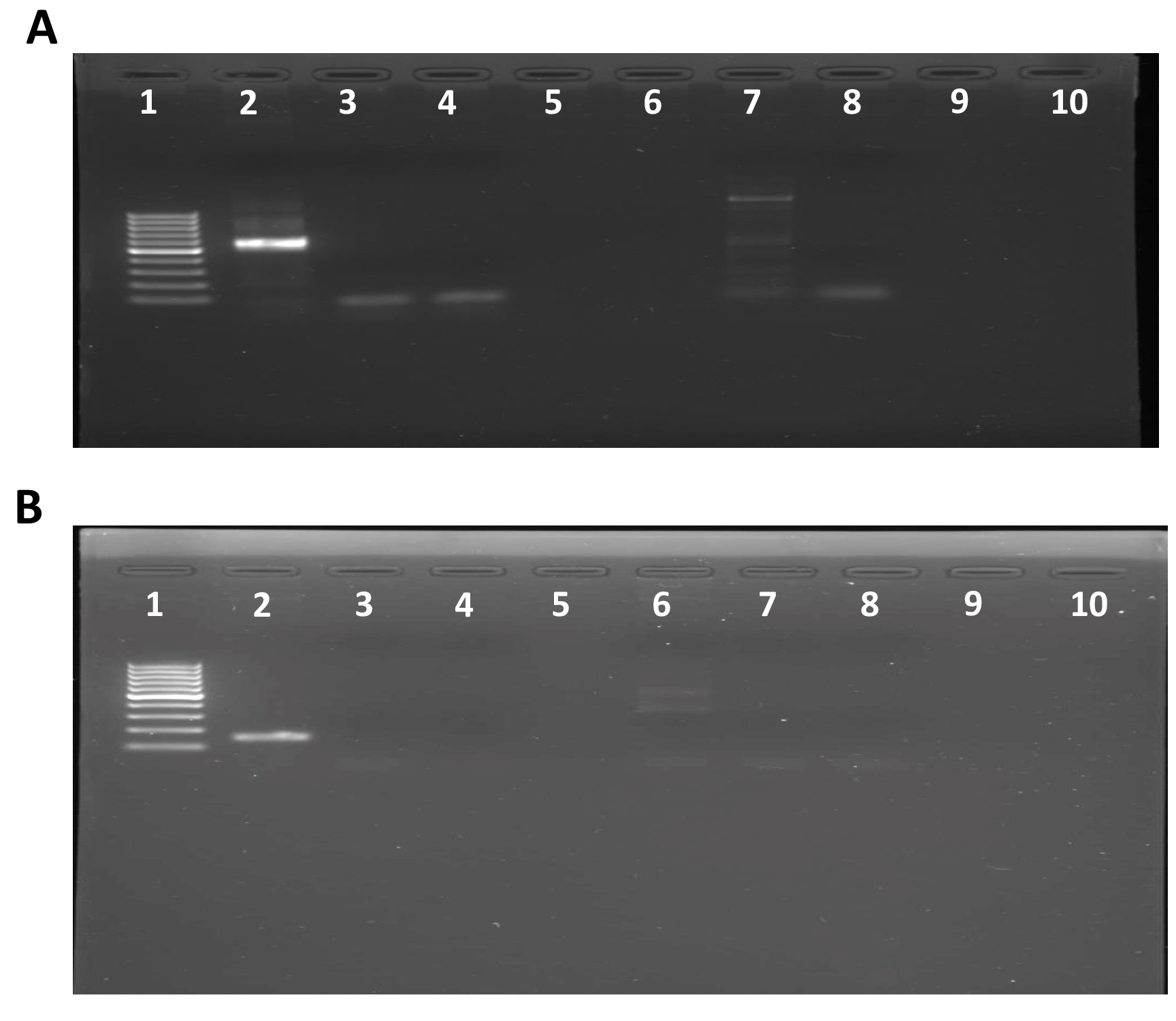

Supplement: Supplementary file 1 — Supplementary Material 1: Supplementary Figure S1 original photograph of the gel electrophoresis of Filarioid PCR products. (A) Amplification of PCR products using filarioid-specific 5.8S-ITS2-28S region primers on a 1.5% agarose gel. Lane 1: GeneRuler 100 bp Plus DNA Ladder; lane 2: dog sample DNA; lane 3: negative control; and lane 4: water control. (B) Amplification of PCR products using primers for the cytochrome oxidase subunit 1 (cox1) fragment specific to D. immitis on a 2% agarose gel. Lane 1: GeneRuler 100 bp Plus DNA Ladder; lane 2: dog sample DNA; lane 3: negative control; and lane 4: water control. Lanes 5 to 10 in both panels (A and B) are not relevant to the current study. [file 12917_2023_3803_MOESM1_ESM.tiff]
